# Sequence-specific detection of different strains of LCMV in a single sample using tentacle probes

**DOI:** 10.1186/s12985-017-0863-9

**Published:** 2017-10-13

**Authors:** Lina S. Franco, Susan A. Holechek, Michael R. Caplan, Joseph N. Blattman

**Affiliations:** 1 School of Molecular Sciences, Arizona State University, Tempe, AZ USA; 2 School of Life Sciences, Arizona State University, Tempe, AZ USA; 3 Simon A. Levin Mathematical, Computational and Modeling Sciences Center, Arizona State University, Tempe, AZ USA; 4 School of Biological and Health Systems Engineering, Arizona State University, Tempe, AZ USA

## Abstract

**Background:**

Virus infections often result in quasispecies of viral strains that can have dramatic impacts on disease outcomes. However, sequencing of viruses to determine strain composition is time consuming and often cost-prohibitive. Rapid, cost-effective methods are needed for accurate measurement of virus diversity to understand virus evolution and can be useful for experimental systems.

**Methods:**

We have developed a novel molecular method for sequence-specific detection of RNA virus genetic variants called Tentacle Probes. The probes are modified molecular beacons that have dramatically improved false positive rates and specificity in routine qPCR. To validate this approach, we have designed Tentacle Probes for two different strains of Lymphocytic Choriomeningitis Virus (LCMV) that differ by only 3 nucleotide substitutions, the parental Armstrong and the more virulent Clone-13 strain. One of these mutations is a missense mutation in the receptor protein GP1 that leads to the Armstrong strain to cause an acute infection and Clone-13 to cause a chronic infection instead. The probes were designed using thermodynamic calculations for hybridization between target or non-target sequences and the probe.

**Results:**

Using this approach, we were able to distinguish these two strains of LCMV individually by a single nucleotide mutation. The assay showed high reproducibility among different concentrations of viral cDNA, as well as high specificity and sensitivity, especially for the Clone-13 Tentacle Probe. Furthermore, in virus mixing experiments we were able to detect less than 10% of Clone-13 cDNA diluted in Armstrong cDNA.

**Conclusions:**

Thus, we have developed a fast, cost-effective approach for identifying Clone-13 strain in a mix of other LCMV strains.

**Electronic supplementary material:**

The online version of this article (10.1186/s12985-017-0863-9) contains supplementary material, which is available to authorized users.

## Background

RNA viruses affect most of the population, especially in developing countries, and are the major cause of emerging and re-emerging diseases in humans [1–3]. One of the most important problems with understanding RNA viruses, including fitness and diversity, is their propensity for rapid change due to its high mutation rate because of lack of proofreading property in the encoded RNA polymerase [3–5]. High mutation rate and high replication rate results in quasispecies even in a single host [4–7] and the presence of quasispecies correlates with enhancement of virulence because of the flexibility of the population of the virus to adapt easily with the dynamics of the host [6]. This phenomenon has been observed in poliovirus, where the presence of quasispecies cooperated the entrance of a neovirulent clone in mice [6], and in evasion of immunity of Hepatitis C Virus [7].

Current diagnostic methods can detect the virus directly, indirectly, fluorescence-based emerging technologies and immune-based. Indirect detection is achieved by detection of virus particles (antigens) or RNA. Fluoresce-based are usually molecular procedures that are used to detect RNA using reverse transcriptase PCR (RT-PCR), which is sensitive and specific, but is highly prone to false-positive results due to easy contamination [8, 9]. The most common molecular technique is using a fluorescent dye that binds to dsDNA and using a calibration curve to quantify the amount of DNA. Another fluorescent-based technique is using probes called molecular beacons, which consist in a stem-loop that, when self-hybridized, places the fluorophore on the 5′ end in close proximity to the quencher at the 3′ end, thus quenching the fluorescence. When the molecular beacon encounters its target, it changes to an open conformation and hybridizes with the target leading to the fluorophore to emit a signal. Tentacle Probes are modified molecular beacons because they have a single stranded DNA attached to the stem-loop by a 9-mer chain of polyethylene glycol (PEG). The ~15 nt single stranded DNA is called capture sequence because, as it is implied by its name, it captures the DNA strand and as a consequence it increases the local concentration of the stem-loop around the DNA strand from a range of nM (free probe) to mM (capture probe bound), increasing the probability of the stem-loop to bind to the target [10, 11]. This technology was successfully used for the detection of *Yersinia pestis* and *Bacillus anthracis* using the bacterial DNA [10], but it has not been tested with any RNA organism or virus.

In this study, we proposed a fast, cost-effective and sequence-specific molecular method for detection of RNA viruses. For this purpose, a virus model for RNA viruses, Lymphocytic Choriomeningitis Virus (LCMV) was used. The method intends to differentiate two strains of LCMV: Clone-13, which causes a chronic infection in mice, versus the wild type Armstrong strain, which causes an acute infection in mice. The difference in type of infection is due to a single mutation in the S segment of LCMV, C855T, which is a missense mutation observed in an amino acid change in the receptor protein GP1 (F260 L). The proposed method uses Tentacle Probes coupled with qPCR and showed that the designed probes to detect Armstrong and Clone-13 strains could differentiate them even with only a single mutation in the RNA that was obtained both in vitro and in vivo. Results also showed that the Clone-13 Tentacle Probe could detect Clone-13 strain with as little as 10% of presence in a sample with a mixture of both strains.

## Methods

### Tentacle probe design

Both Tentacle Probes were designed based on the generalizations of the principles described by Satterfield et al. [11]*.* Briefly, four hundred possible combinations of detection probes and capture probes were simulated in a Microsoft Excel file to obtain a suitable probe to detect the strain Clone-13 of LCMV over the Armstrong strain, and vice versa for the detection of Armstrong strain. The sequences of the possible combinations were made adding one or more nucleotides to the complementary sequence around a 10 nt sequence, which included the target mutation site, either downstream or upstream. DINAMelt and UNAFold applications [12, 13] were used to calculate melting temperatures of the detection probe stem, the capture probe-target duplex, and the detection probe-target duplex, independent of each other. The targeted single mutation was located in the loop of the detection probe. The detection and capture probes were designed with predicted melting temperatures around the predicted assay temperature of 60 °C. The melting temperature for the stem-loop structure was chosen 7–10 °C above the assay conditions (70 °C). The most appropriate probe from the simulated probes with the best differentiation in fluorescence prediction between target and non-target was purchased from Biosearch Technologies as a custom oligonucleotide to use for the detection of each strain (Biosearch Technologies, Petaluma, CA, https://www.biosearchtech.com). The calculations obtained for each probe are summarized in Table 1.

**Table 1:** Probe sequences for each strain and their thermodynamic parameters calculated. Equilibrium constants and ratio of probe predicted to fluoresce for each probe

Probe sequence	K_stemloop_	K_detectionT_ (μM^−1^)	K_captureT_ (μM^−1^)	Fraction of fluorophore predicted to be fluorescing with Target	Fraction of fluorophore predicted to be fluorescing with Non-Target
*Clone13:* FAM-cgtaagTTCCTCTCACGaacttcg-BHQ-PEG9-acattcacctggactttgtcagactc	62.35	82.53	1.43 × 10^8^	0.5447	0.0053
*Armstrong:* FAM-cgtagtGATTCTTCACtacg-BHQ-PEG9-agcgggcacattcacctgg	26.44	33.93	6.9 × 10^9^	0.3188	0.0276

### RNA isolation and cDNA obtention

#### a. RNA isolation from BHK21 cells cell virus collection

RNA isolation was performed following the guanidinine thiocyanate-phenol-chloroform (GNTC) procedure described before [14] with some modifications. Briefly, virus supernatant of each strain was mixed with GNTC in a 1:2 ratio, followed by the addition of 0.1 volumes of 2 M sodium acetate pH = 4, 1 volume of Acid phenol pH = 4.3 and 0.3 volumes of chloroform-isoamyl-alcohol 24:1 (Sigma Aldrich Inc., St. Louis, MO, http://www.sigmaaldrich.com/united-states.html). Samples were centrifuged at 20000×*g* for 30 min at 4 °C, then the aqueous phase was transferred into a new tube and 1.1 volumes of isopropanol were added and left at −20 °C overnight. Next, samples were centrifuged at 20000×*g* for 30 min at 4 °C, supernatant was discarded and 1 volume of 70% ethanol was added. Another centrifugation step at the same conditions was performed, after which the supernatant was removed completely, the tube was air-dried and the pellet was resuspended in nuclease-free water. All RNA concentrations were measured using Nanodrop 1000. cDNA was obtained in a two-step procedure with Omniscript RT kit (QIAGEN, Valencia, CA, https://www.qiagen.com/us/) and posteriorly amplifying a 538 nt amplicon of the S segment of LCMV that includes the C855T mutation found in Clone-13 with PCR using Phusion® High-Fidelity DNA polymerase (New England Biolabs, Ipswich, MA, https://www.neb.com) to obtain a high concentration of target and non-target samples. The primers used are the following: Forward: 5’AGCCAGTGTAGAACCTTCAGAG3’ and Reverse: 5’AGTGGTTCCTCATCAGTAGTTG3’. The cycling conditions for the PCR were the following: 30 s at 94 °C, 30 cycles of 20 s at 94 °C, 30 s at 53 °C and 2 min at 72 °C, and a final extension of 5 min at 72 °C.

#### b. RNA isolation from plaques

Three serum samples from infected mice with a LCMV (unknown strain) were obtained from a previous study. All studies were conducted according to animal protocol 12-1229R under the approval and guidance of the Arizona State University Institute for Animal Care and Use Committee. A plaque assay was performed as previously described [15] with the sera and RNA from plaques was obtained mixing SM buffer with the extracted plaques and letting them sit overnight at 4 °C. Then, RNA was isolated using the RNAqueous® Total RNA Isolation Kit (Thermo Fisher Scientific, Waltham, MA, https://www.thermofisher.com/us/en/home.html). All RNA concentrations were measured using Nanodrop 1000. cDNA was obtained through RT-PCR performed with SuperScriptIII One step RT-PCR system with Platinum® Taq DNA polymerase (Thermo Fisher Scientific, Waltham, MA, https://www.thermofisher.com/us/en/home.html). Primers used were the same used for the PCR described above to generate cDNA. The cycling conditions were the following: 30 min at 50 °C, 2 min at 94 °C, 35 cycles of 15 s at 94 °C, 30 s at 53 °C and 2 min at 68 °C, and a final extension of 5 min at 68 °C. The samples were also sent for sequencing with either of the primers to confirm the qPCR and Tentacle Probes’ result.

### Melting curve and qPCR

**Fig. 1:**
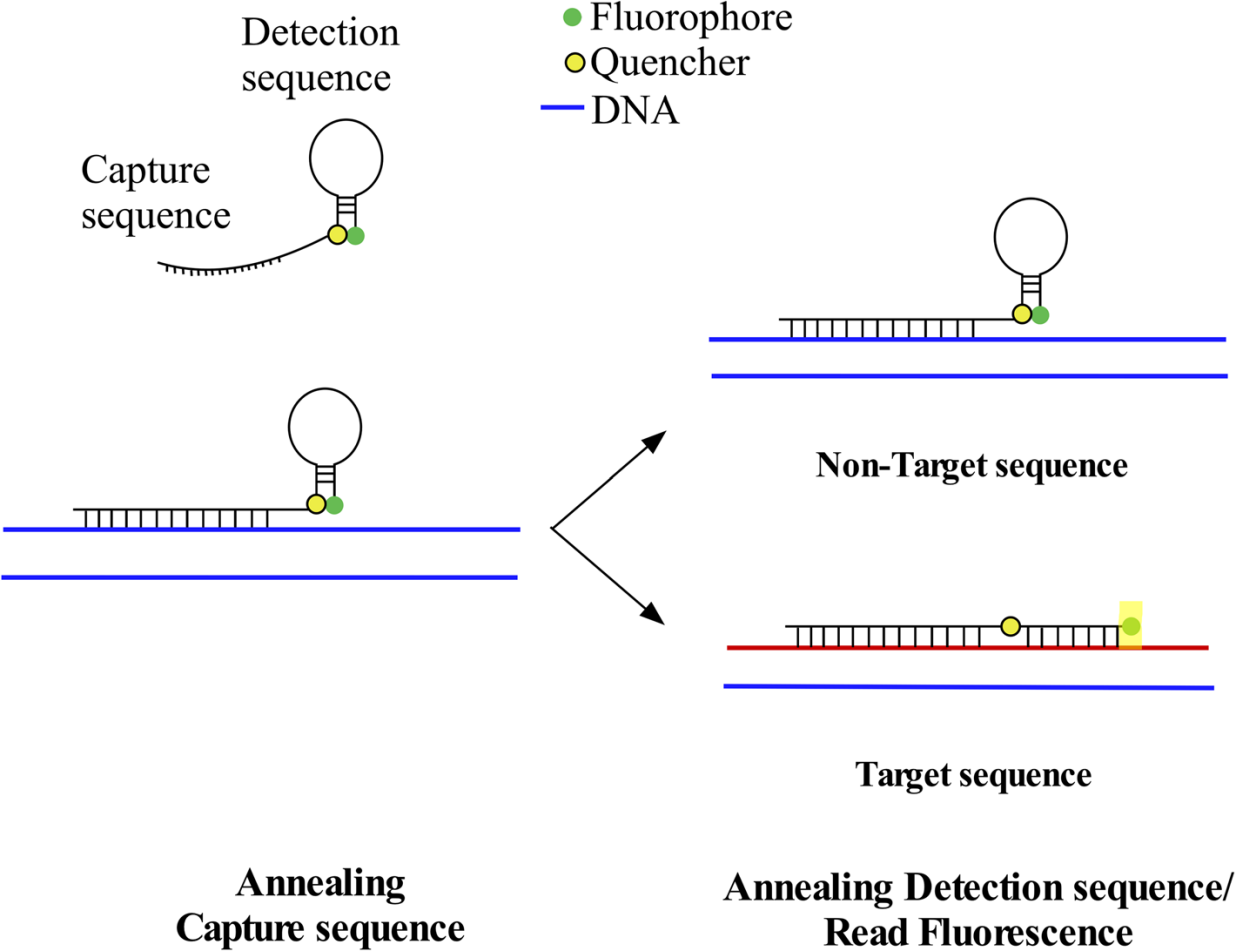
Tentacle probe mechanism of detection of the target. In the annealing step, the capture sequence of the Tentacle Probe hybridizes to the DNA nearby the target sequence to allow proximity of the detection sequence to the target sequence. The detection sequence hybridizes the target sequence enabling the fluorophore to separate from the quencher and read the fluorescence. In the case of a non-target sequence, the detection sequence will not hybridize and consequently the fluorophore will not separate from the quencher. Adapted from [10]

The melting curve from the probe was obtained to determine the temperature at which the target and non-target obtained from BHK21 viral cDNA were best differentiated in the qPCR. To a 20 μl reaction with 10 μl of the designed tentacle probe (final concentration: 50 nM) and 10 μl either 1 nM of target or non-target DNA, fluorescence at 10 °C until 90 °C every 0.6 °C/s was measured. For the qPCR, the primers were designed to obtain a 150 nt amplicon of the S segment of LCMV that contained the C855T mutation found in Clone-13 strain compared to Armstrong strain, and are the following: Forward: 5’CAGGTCCTTTTGGGATGTCCAG3’, Reverse: 5’CTCTGCAGCAAGAATCATCCATTTG3’. For these experiments, a 20 μl reaction included 1.5 U/rxn of Platinum® Taq DNA polymerase, 0.1 μM of the tentacle probe, 0.5 μM of each primer, 5 mM (Clone-13) or 1.5 mM (Armstrong) MgCl_2_, 1X Buffer, 0.2 mM dNTPs and 0.25 nM of cDNA (sample, target or non-target) for all the experiments except for the optimization experiments where some of these concentrations (MgCl_2_, cDNA) vary. The cycling conditions for the qPCR were the following: 2 min at 95 °C, 40 cycles of 1 s at 90 °C and 3 min at 50 °C. The mechanism is illustrated in Fig. 1. These conditions were standardized for each probe as shown in results. qPCR was performed in a ViiA 7 Real-Time PCR System from Life technologies (Thermo Fisher Scientific, Waltham, MA, https://www.thermofisher.com/us/en/home.html). Each sample was tested at least in triplicate. Raw fluorescence counts per cycle were further analyzed for comparison between samples. The proposed threshold to consider a sample positive was set to be the average of the non-target controls plus two standard deviations $\left(\overline{X_{NT}}+2{\sigma}_{NT}\right)$.

### PCR for differentiation between mutants

As a comparison to the proposed method, we used a specific primer containing the mutation to differentiate between the strains. Samples used for this experiment was viral cDNA obtained from BHK21 supernatant as described above. The primers used for detection of Armstrong strain are the following: Forward: 5’AGCCAGTGTAGAACCTTCAGAG3’ and Reverse: 5′ AGTCTCCTAGTGAAGAACTTAG3’. In the case of Clone-13 the primers used were the following: Forward: 5’AGCCAGTGTAGAACCTTCAGAG3’ and Reverse: 5′ AGTCTCCTAGTGAGGAACTTAG3’. This experiment was performed using Phusion® High-Fidelity DNA polymerase (New England Biolabs, Ipswich, MA, https://www.neb.com) with the following cycling conditions: 30 s at 94 °C, 30 cycles of 20 s at 94 °C, 15 s at 53 °C and 1 min at 72 °C, and a final extension of 2 min at 72 °C. The resulting 248 bp fragment was observed in a 3% agarose gel using SYBR Gold Nucleic Acid Stain (Thermo Fisher Scientific, Waltham, MA, https://www.thermofisher.com/us/en/home.html).

## Results

The differentiation of strains or genotypes of viruses can determine and, in many cases, predict the virulence of an infection. In the case of Dengue, for example, it is important to determine the serotype as a method of surveillance in the population to try to prevent hemorrhagic fevers [16, 17]. In the case of LCMV, a different strain or genotype can determine the type of infection: chronic (Clone-13) or acute (Armstrong). For this purpose, a tentacle probe was designed to detect Clone-13 over Armstrong and vice versa. The thermodynamic parameters obtained using DINAMelt and UNAFold applications [12, 13] were used to calculate the equilibrium constants for each one of the hybridization steps of the capture and detection probe described by Satterfield et al. [11], to predict the fraction of probe fluorescing with the target or non-target. These predictions helped determine the most suitable combination of detection and capture sequence among more than ~400 possible sequences that were simulated.

For the probe designed to detect Clone-13 strain, the calculations predicted that the ratio of probe fluorescing with the target would be 54.47% versus 0.53% with the non-target (Table 1). For the probe designed to detect the Armstrong strain the percentages were 31.88% and 2.76% with the target vs the non-target, respectively. With the thermodynamic calculations it could also be determined the increase in the equilibrium constant when it takes into account the hybridization of the capture sequence (Table 1), rather than when it is not present in the probe, as in molecular beacons.

### Melting curve and optimization of conditions for qPCR

**Fig. 2:**
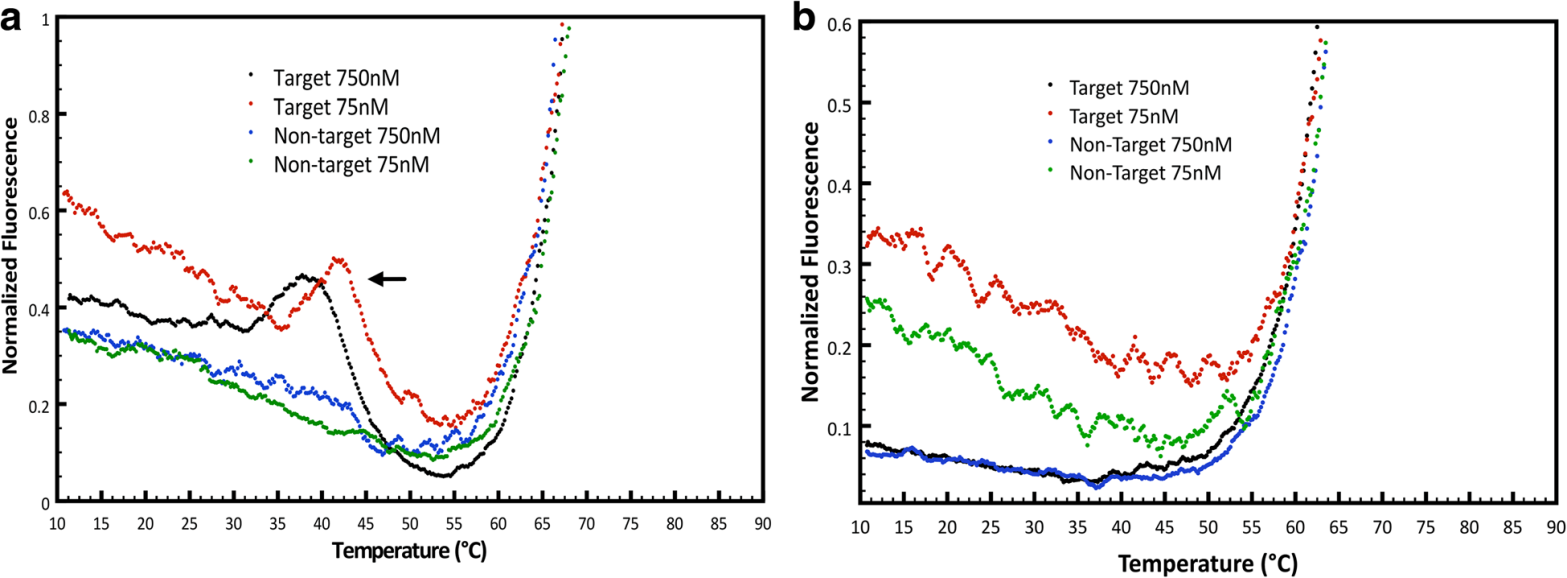
Melting curve to confirm the annealing temperature to be used. Determination of the optimal annealing temperature with a melting curve of the **a**) Clone-13 detection probe and **b**) Armstrong detection probe, to differentiate between target and non-target with a melting curve ran at 75 and 750 nM of the initial sample target or non-target DNA

**Fig. 3:**
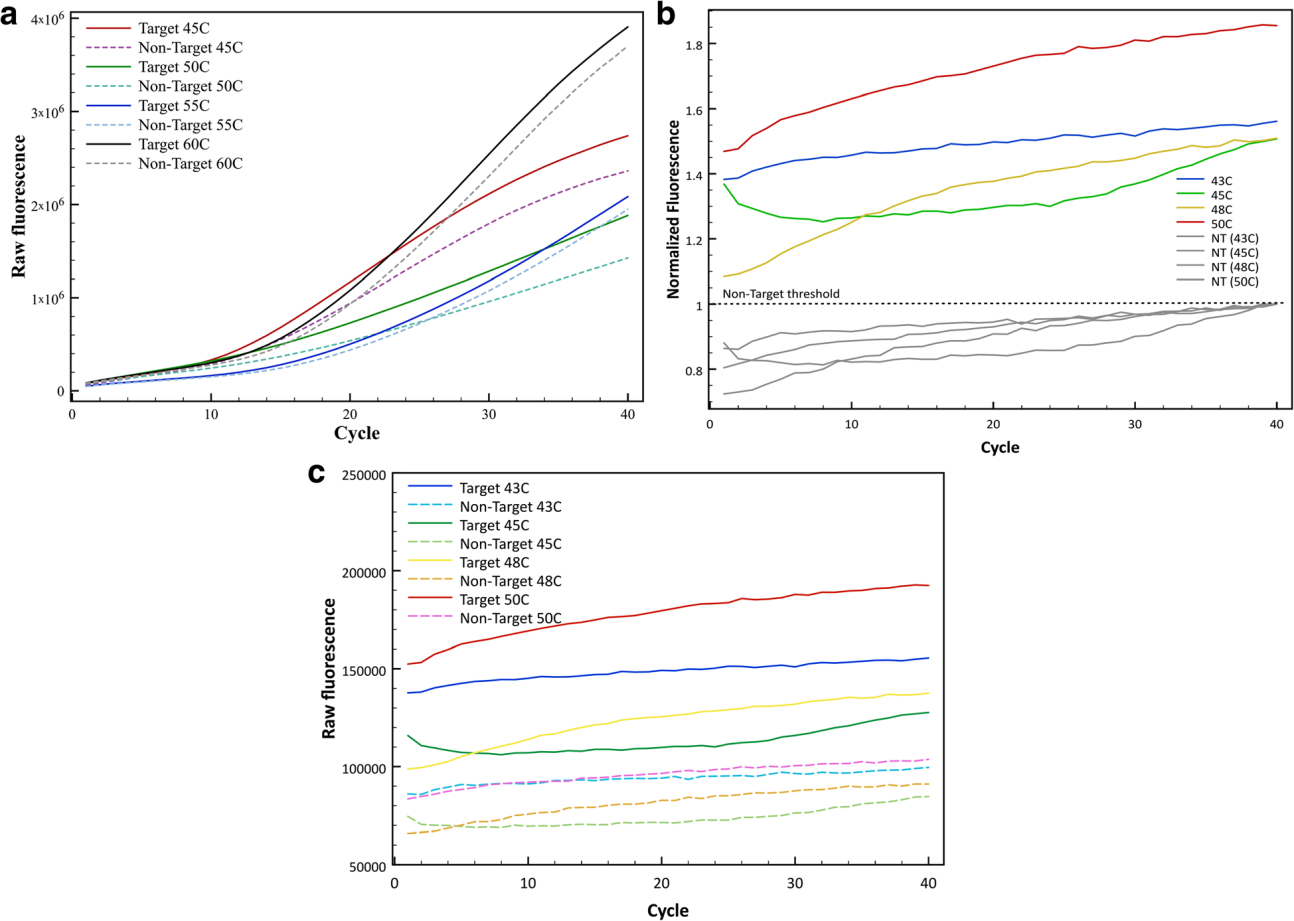
Optimization of annealing temperature for Clone-13 probe and Armstrong probe. **a** Raw fluorescence measured at different annealing temperatures (45, 50, 55 and 60 °C) of Clone-13 (solid lines) and Armstrong DNA (dashed lines) with the Clone-13 probe. **b** Normalized Fluorescence to the maximum non-target fluorescence at different annealing temperatures (43, 45, 48 and 50 °C) of Armstrong (color lines) and Clone13 DNA (gray lines) with the Armstrong probe, and **c**) Raw fluorescence counts at the same annealing temperatures of Armstrong (solid lines) and Clone-13 DNA (dashed lines) with the Armstrong probe

To confirm the overall melting temperature calculated by DINAMelt and UNAFold of each one of the probes, a melting curve was performed at different DNA concentrations obtained from harvesting virus in BHK21 cells for the target and the non-target in each case. Although the thermodynamic calculations are a good estimate to determine the best differentiation, the prediction is not accurate because as observed in the melting curve for both probes (Fig. 2), the detection of the non-target was considerably higher than 0.053% and 2.76%, respectively. Figure 2a shows that the best range at which the Clone-13 probe differentiates the target (Clone-13) and non-target (Armstrong) DNA is around 40 °C, but annealing temperatures in PCR are known to be best in the 45-60 °C range. For this purpose, qPCRs were performed at 4 different annealing temperatures along this range: 45, 50, 55 and 60 °C. Figure 3a shows raw fluorescence counts of the target (solid lines) and non-target (dashed lines) at each annealing temperature with the Clone-13 probe. After each cycle, it is observed how raw fluorescence is increasing Although at each temperature there is a visible difference between target and non-target, the best differentiation at maximum fluorescence was observed using an annealing temperature of 50 °C with ~1.3 fold of maximum fluorescence.

For the Armstrong probe, Fig. 2b shows the melting curve for this probe and it suggests that the best range at which it can differentiate best target (Armstrong) and non-target (Clone-13) DNA is between 40 and 50 °C, but practical limits on cooling make those impractical and amplification is less reliable at lower temperatures. Four different qPCRs were performed with different annealing temperatures along this range: 42, 45, 48 and 50 °C, and were evaluated for the best differentiation between target and non-target. Figure 3b shows normalized fluorescence counts to the maximum non-target fluorescence at each temperature tested and Fig. 3c shows raw fluorescence counts for the same temperatures. Both graph suggest that the best annealing temperature for this probe is 50 °C, showing ~1.85 fold of maximum fluorescence for the target versus non-target. It is important to mention that for this probe it is not observed the exponential phase in the fluorescence curves, as it is expected in a qPCR curve, in both raw and normalized fluorescence counts (Fig. 3b and c).

For the annealing temperature, all the thermodynamic calculations were based in an assay temperature of 60 °C for each probe, although as observed with the melting curve for each probe (Fig. 2), the melting temperature for each was lower than expected, and by 60 °C the probe was completely dissociated for both the target and the non-target. The best annealing temperature for each probe was chosen because it gave the maximum differentiation between target and non-target at the same DNA concentration.

After determining which was the optimal annealing temperature to differentiate target and non-target DNA, two other parameters were optimized: the optimal initial sample DNA concentration for each reaction and the MgCl_2_ concentration needed in the reaction. The concentration of MgCl_2_ is crucial for the activity of the enzyme and even the salt effect on DNA. Three or four concentrations (0.5, 1.5, 3 and 5 mM) were tested for best results, including the concentration suggested to use by the manufacturer of the enzyme employed. Results showed that for the Clone-13 probe the most suitable concentration to use was 5 mM with ~1.4 fold maximum fluorescence of the target vs non-target, and for Armstrong probe, the most suitable concentration to use was 1.5 mM with ~1.5 fold maximum fluorescence of the target vs non-target (Additional file 1: Figure S1).

**Fig. 4:**
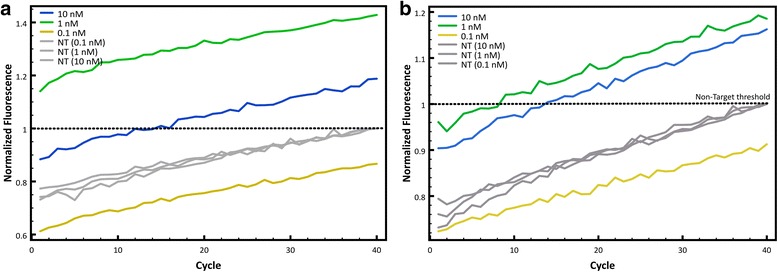
Optimization of minimum starting concentration of DNA in sample. **a** Fluorescence counts of Clone13 (color lines) and Armstrong DNA (gray lines) with different initial DNA concentrations. **b** Fluorescence counts of Armstrong (color lines) and Clone13 DNA (gray lines) with different initial DNA concentrations

**Fig. 5:**
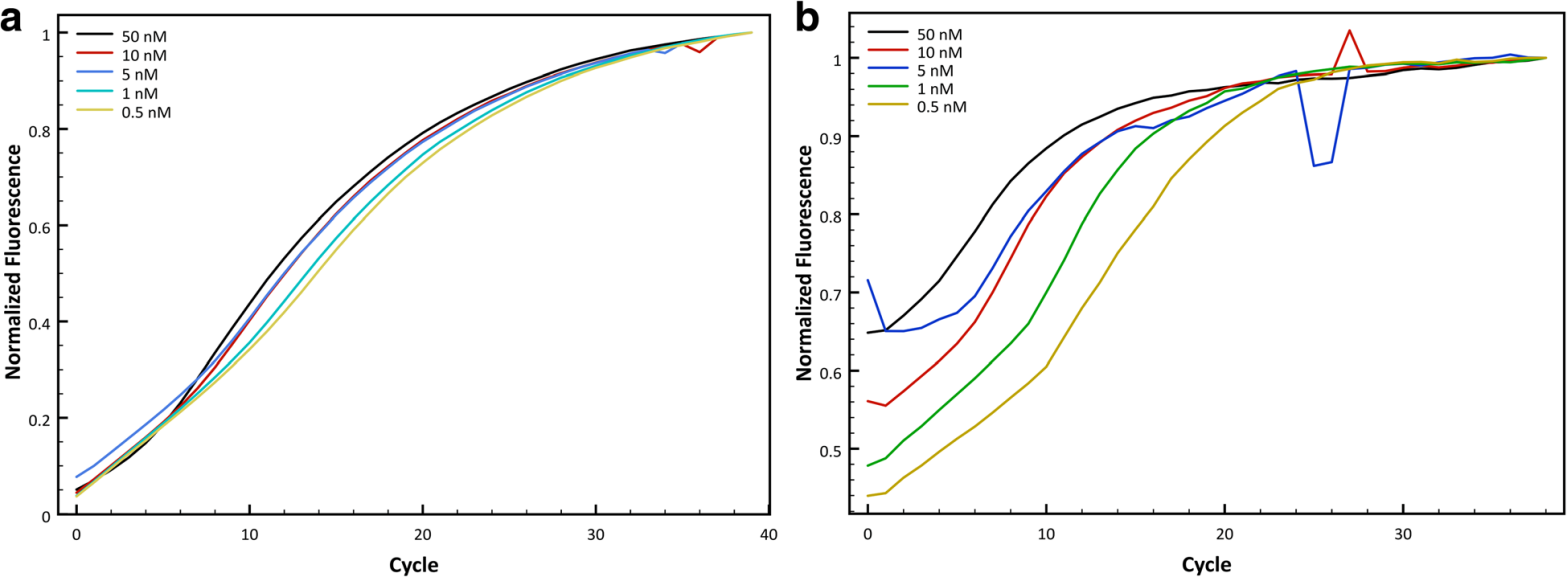
Reproducibility of the assay. Different concentrations of viral cDNA (0.5, 1, 5, 10, and 50 nM) were tested with the Tentacle Probes to show reproducibility of the assay. The curve shown is the average of 30-35 replicates. **a** Test with Clone-13 probe, **b** Test with Armstrong probe

Finally, to determine the minimal concentration at which the fluorescence is best for target detection, three initial DNA concentrations were evaluated: 0.1, 1 and 10 nM. This experiment help estimate the minimal amount of DNA needed so the probe can differentiate between target and non-target. Figure 4a shows the normalized fluorescence counts obtained with different starting concentrations of sample DNA for the Clone-13 probe and Fig. 4b shows the same but with the Armstrong probe. Both graphs show that differentiation between target and non-target is best when using a sample concentration of 1 nM as a starting DNA concentration. Although 10 nM of DNA also showed good results, the best fluorescence differentiation between target and non-target is using 1 nM of sample DNA. To show reproducibility of the assay, different concentrations of viral cDNA were tested with each probe and 30-35 replicates were run. The minimum concentration (0.5 nM) was chosen according to the limit of detection of the qPCR, which for the fragment amplified is ~0.3 nM. Figure 5 shows that for both probes after 15 cycles, the curves of all concentrations would reach a measurable fluorescence. For the Clone-13 probe there is almost no difference between different concentrations even at low concentrations. For the Armstrong probe, at lower concentrations the C_T_ shifts to a higher cycle due to the initial amount of DNA.

### Determination of specificity and sensitivity for each probe

Once the conditions for the probes were optimized, a blind experiment was performed to determine the specificity of each probe. The experiment consisted in measuring the fluorescence by qPCR of 30 samples, 15 target and 15 non-target, that were organized randomly by another scientist, who knew the order of these samples. With the fluorescence results obtained, the samples were assigned as target or non-target compared to a positive and negative control, and afterwards the assignment was compared to the real identity of the samples. A sample was assigned as target if the maximum normalized fluorescence was higher than a threshold: the mean of the non-target maximum normalized fluorescence plus two standard deviations $\left(\overline{X_{NT}}+2{\sigma}_{NT}\right)$. This was defined as the statistically significant threshold or limit of detection between target and non-target. The experiment was performed in triplicate for each probe. For the Clone-13 probe, 29 of 30 samples were assigned accurately: 14 true positives, 1 false negative, 15 true negatives, and 0 false positives (Additional file 2: Figure S2A). For the Armstrong probe, 25 of 30 samples were assigned accurately: 13 true positives, 2 false negatives, 12 true negatives, and 3 false positive (Additional file 2: Figure S2B).

### Quasispecies detection

**Fig. 6:**
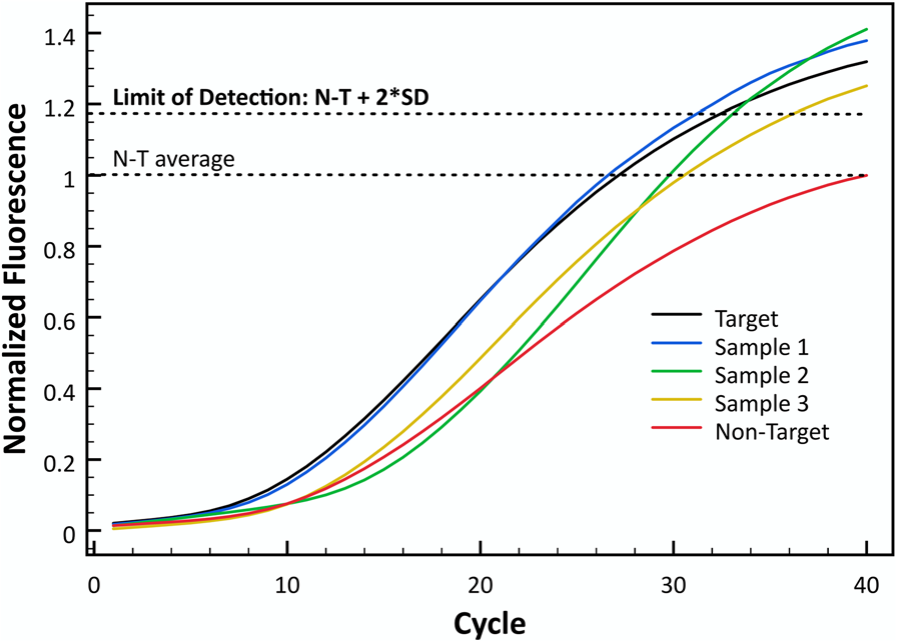
Quasi-species test of three serum samples obtained from mice. Serum from infected mice with an unknown LCMV strain was tested to detect Clone-13 with the correspondent probe. The dashed line represents the non-target threshold proposed $\left(\overline{X_{NT}}+2{\sigma}_{NT}\right)$

After the conditions of the qPCR for the probes were optimized and it was shown that the probes could detect specifically each strain in samples with mixtures, serum samples from mice infected with an unknown strain of LCMV, suspected to be Clone-13, were tested to detect Clone-13 or Armstrong strain. Plaque assay was performed with these samples and RNA was isolated from plaques from each sample in triplicate. qPCR with the Clone-13 probe was performed to test for the presence of this strain in the samples. Figure 6 shows the normalized fluorescence of the three samples tested compared to a positive control (target) and a negative control (non-target). It is observed that the maximum fluorescence for the three samples was above the proposed threshold in previous experiments $\left(\overline{X_{NT}}+2{\sigma}_{NT}\right)$. To compare the results with other detection method, the DNA obtained from the samples was sequenced and confirmed the results obtained with the tentacle probe for Clone-13 detection.

### Detection of a single mutation difference in a mixture of strains

**Fig. 7:**
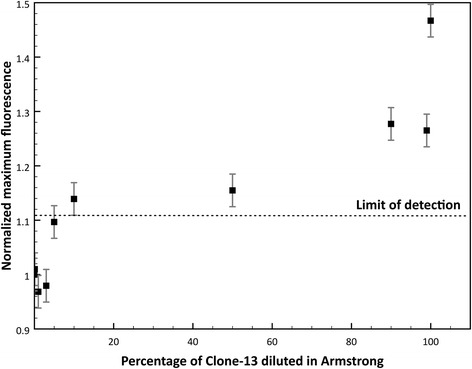
Determination of the limit of detection of the Clone-13 probe. The probe designed to detect Clone-13 is diluted in Armstrong by combining different proportions of Clone-13 (target) and Armstrong (non-target) DNA

Detection of specific viruses even between the same family is essential, especially in clinical samples where samples sometimes present more than one viral infection at a time or can present different quasispecies in the same host. In nature LCMV wild type strain Armstrong is commonly found, but Clone-13 strain is not found frequently even though it causes chronic disease in rodents. To further test the lower limit of detection of Clone-13 strain in a mixture of the other strain (Armstrong), mixtures of target and non-target in different proportions were tested to detect Clone-13 strain using its specific probe. Figure 7 shows the normalized maximum fluorescence of the mixtures of Clone13 and Armstrong samples ranging from 0.1-100% of Clone-13 DNA. The dashed line represents the limit of detection proposed for the blind experiment $\left(\overline{X_{NT}}+2{\sigma}_{NT}\right)$. It is observed that the probe can detect to a lower limit of detection of approximately 10% of the Clone-13 when it is present in a mixture with Armstrong DNA.

## Discussion

Fast and cost-effective detection methods for viruses are currently needed for opportune diagnosis and treatment of viral disease. In this study, we tested the specificity of Tentacle Probes to detect one strain of LCMV that only differs from the other strain in three point mutations, specifically one missense mutation in the S segment. The mutation that was studied causes a change in one amino acid of the receptor protein GP1, which also changes the type of infection induced (chronic vs. acute) and is the main reason why we chose this mutation above the other two mutations present compared with the wild type. The design of the probe for the detection of each strain was following the calculations proposed by Satterfield et al. [11] In here, the calculated thermodynamic parameters (i.e. enthalpy, entropy) with DINAMelt application were used to calculate the equilibrium constants and therefore estimate the probe fluorescing when is bound to the target and non-target (Table 1). The predicted amount of probe fluorescing for both probes did not agree as precisely with what was observed. For the Clone-13 probe, the predicted difference in fluorescence between the target and the non-target was 50% higher, while the fluorescence obtained experimentally was ~40% higher, on average. A similar behavior was observed with the probe designed to detect Armstrong strain. This can be attributed to the fact that the predicted calculations did not take into account any kinetic factors, but only thermodynamic. Kinetic factors, like size of the probe, not only can be as important as the thermodynamic ones, but also can define entirely how much time a step in the qPCR might need in order to achieve the conformation desired in the case of the stem-loop (open or closed) [11, 18]. Nevertheless, the predicted fluorescence of the probe bound to the target or non-target is a good guide to choose the most suitable combination of capture and detection probe that will favor hybridization with the target.

Validation of the method was described in detail by optimizing the qPCR conditions for each probe, including annealing temperature, MgCl_2_ concentration and starting sample DNA concentration (Figs. 3, 4 and Additional file 1). The annealing temperature was expected to be higher than the melting temperature of the individual regions (capture and detection probes) because the principles of cooperativity indicate that the affinities of each part of the probe should be slightly weaker than the affinity required to achieve binding of the whole probe. However, as mentioned before, calculations did not take into account important kinetic factors such as the size of the whole probe and each part in separate [18] that might have affected the variance in the experimental annealing temperature compared to the predicted temperature. It is important to mention that the curves observed with the Armstrong probe at all temperatures did not show the exponential region (sigmoidal shape) usually observed with qPCR curves, as it was observed with the Clone-13 probe. This is an indication that the Armstrong probe is not as fit as the Clone-13 probe and is in agreement with the thermodynamic predictions of fluorescence with the target and the non-target, where the differentiation with the Armstrong probe is not as pronounced as the differentiation predicted with the Clone-13 probe (Table 1).

Reproducibility of the assay was observed for different concentrations of each target with the correspondent probe. Clone-13 probe showed high sensitivity at all concentrations compared to the Armstrong probe, which had a higher C_T_ at lower concentrations (1 and 0.5 nM). However, it is important to highlight that for the Armstrong probe one of the false negatives seems to not have been amplified the amplicon at all (purple flat line). This error could be due to other experimental errors (i.e. some mistake in the master mix preparation) rather than a lack of specificity of the probe because each unknown had only one replicate. As observed with the other experiments, this experiment also reflects the strength of both of the probes, which confirmed the initial predictions of the thermodynamic calculations.

MgCl_2_ concentration is another important factor because it has a high contribution in ionic strength of the reaction and therefore influence directly into the thermodynamics. The calculations were performed to use 5 mM of MgCl_2_ and 1.5 mM for the Clone-13 and Armstrong probe, respectively. The concentration used for the calculation of the Clone-13 probe was the one recommended by Satterfield et al. and the concentration used for the calculations of the Armstrong probe was the one recommended by the manufacturer to use for an optimal reaction using Platinum Taq polymerase. There was an agreement of the expected optimal concentration of MgCl_2_ with the one observed for both the Clone-13 probe and Armstrong probe. This shows that the ionic strength used in the calculations accurately predicted the optimal salt concentration in the reaction.

It was observed that the calculations showed a better differentiation between target and non-target for the Clone-13 probe than for the Armstrong probe (Table 1). The difference between the two concentrations of MgCl_2_ for the calculations might be one of the factors for the lower efficiency of the Armstrong probe compared to the Clone-13 probe. This difference in efficiency of detecting one strain or the other can also be attributed to the thermodynamic advantage of the Clone-13 probe due to the presence of the cytosine in the mutation site, instead of the thymine like in Armstrong probe. Cytosine will hybridize with a guanine forming three hydrogen bonds, meaning the base-pairing hybridization will be more stable than the hybridization of the mismatch (anomalous cytosine-adenine pairing), which means that the hybrid between the probe and the target (C-G) is favored by stability. For the Armstrong probe, the thymine from the probe will hybridize with the adenine forming only two hydrogen bonds with the target instead of the hybrid with the non-target mismatch (anomalous thymine-guanine pairing), in which case it would form three hydrogen bonds. Although the probability of the hybridization of the Armstrong probe with the mismatch is low, due to the necessary conversion from the keto-thymine to the enol-thymine conformation, the hybridization of thymine and adenine (Armstrong probe-target) is less favored thermodynamically than the hybridization guanine-cytosine (Clone-13 probe-target), in terms of Gibbs free energy.

Experimentally it was shown that each probe designed could differentiate effectively one strain from the other. The blind tests performed with each probe to determine the specificity and sensitivity gave a sense of how accurate the probe could detect the target strain. The Clone-13 probe showed a better specificity giving results with only 6% of false-negatives. On the other hand, the Armstrong probe showed results with 13% of false-negatives and 20% of false positives. It was expected that the Armstrong probe would have a higher probability of obtaining an inaccurate result, due to the smaller difference of expected fluorescence with the target vs. non-target in the initial calculations. Nevertheless, these percentages could be lower and further experiments with a bigger sample size (>50 samples) could be used to determine the percentage of false-positives and false-negatives more accurately.

This method reduced false-positives compared to common real time PCRs used for other RNA viruses such as Dengue [8] and common PCRs with a difference of only one point mutation between strains, as observed when using specific primers to detect each strain (Additional file 3: Figure S3). The reduction of false-positives with this method is an advantage when using it with clinical samples overall in tropical regions, where many viruses with similar symptoms affect the same population (i.e. Dengue and Zika virus) [16, 19]. Furthermore, detection of viral strains in serum using this method was established when we could accurately detect Clone-13 virus in the serum samples from infected mice (Fig. 6).

Also, the probe tested could effectively differentiate each strain in virus mixing experiments with the two strains. Clone-13 probe can detect down to 10% of Clone-13 diluted in Armstrong (Fig. 7). The detection of one strain in a mix of other strains of viruses has a high impact in a detection method because it simulates the conditions of many clinical samples. This experiment in particular intended to illustrate two important viral phenomena: the presence of quasispecies in the population of viral strains, even in the same host, and co-infection of two viruses in one host. Infections with RNA viruses can often result in the formation of quasispecies of virus strains that can rapidly adapt and can develop resistance to anti-viral drugs or vaccines [4]. Quasispecies of viral strains differ between them in a few mutations, even in only one mutation [2, 4, 6, 20]. Accurate detection of one of these quasispecies over another one can be crucial to determine possible treatment of the infection, the pathogenicity, and the evolution of a determined virus in a population. Another scenario where detection of one viral strain in a mixed sample is essential is in viral co-infections. Co-infection of two unrelated viruses that affect the same population is very common in the “real world” [21]. The presence of two or more viruses in a host can modulate the immune response and/or alter the disease [21, 22]. Therefore, the use of Tentacle Probes and qPCR for the detection of RNA viruses can be widely applied in many situations where high specificity and reduction of false-positives is needed.

## Conclusions

Herein, we showed that each Tentacle Probe could detect with specificity each strain studied in samples with a single strain, and, as low as 10% of the target, in samples with a mixture of strains. The thermodynamic calculations performed to determine the most suitable sequence of the probe to detect the target vs the non-target, were accurate to establish the necessary ionic strength and starting DNA sample concentration, but not as accurate in terms of the fluorescence detected by each probe. Nevertheless, the calculations are a good guide to determine the best detection and capture sequence to use.

Although currently there are other methods to detect quasispecies like sequencing, they are still cost-prohibitive. This study describes a fast cost-effective detection method that was designed for differentiating RNA viruses, even between close related viruses or quasispecies of a virus. This method could potentially be useful not only to measure virus diversity, in terms of measuring absence/presence of known variants, but to understand virus evolution, pathogenicity of the virus, and to determine possible treatment of infection.

## Additional files

Additional file 1: Figure S1. Optimization of MgC1_2_ concentration in the qPCR reaction. A) Fluorescence counts of Clone13 (color lines) and Armstrong DNA (gray lines) with different MgC1_2_ concentrations. B) Fluorescence counts of Armstrong (color lines) and Clone13 DNA (gray lines) with different MgC1_2_ concentrations. (PDF 1739 kb)

Additional file 2: Figure S2. Sensitivity and specificity. Thirty samples of viral cDNA were subject to test if they were target or non-target depending on the Tentacle Probe used compared to a negative and a positive control. A) Test for Clone-13 Tentacle Probe, B) Test for the Armstrong Tentacle Probe. Yellow lines represent false positive, purple lines represent false negative, red line is the average of the negative control, black line is the average of the positive control. True positive are in blue and true negatives are in gray. (TIFF 4096 kb)

Additional file 3: PCR detection of mutants with specific primers for each strain. Detection of each specific strain is observed in lane 2 and 4, although unspecific bands are also observed when the primers are exposed to the other strain (lane 3 and 5), which is different in only one nucleotide. (TIFF 1377 kb)

## Abbreviations

BHK21: Baby hamster kidney cells
CDNA: Complementary DNA
GNTC: Guanidinine thiocyanate-phenol-chloroform
LCMV: Lymphocytic choriomeningitis virus
PCR: Polymerase chain reaction
QPCR: Quantitative polymerase chain reaction
RT-PCR: Reverse transcriptase polymerase chain reaction
