# Supplementary material for: Sequence-specific detection of different strains of LCMV in a single sample using tentacle probes
Source: Virol J. 2017 Oct 13;14:197. doi: 10.1186/s12985-017-0863-9 (PMC5640925; doi:10.1186/s12985-017-0863-9)
Supplement: Supplementary file 1 — Optimization of MgC12 concentration in the qPCR reaction. A) Fluorescence counts of Clone13 (color lines) and Armstrong DNA (gray lines) with different MgC12 concentrations. B) Fluorescence counts of Armstrong (color lines) and Clone13 DNA (gray lines) with different MgC12 concentrations. (PDF 1739 kb) [file 12985_2017_863_MOESM1_ESM.pdf]

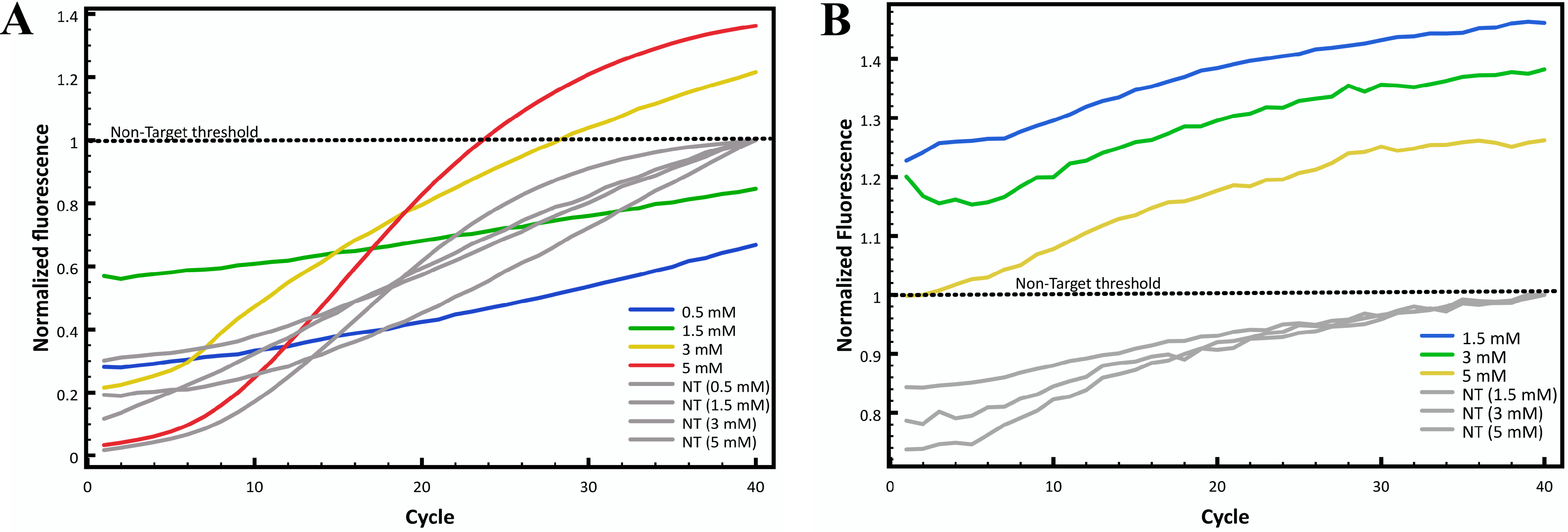

S1 Figure 1. Optimization of $MgCl_2$ concentration in the qPCR reaction. A) Fluorescence counts of Clone13 (color lines) and Armstrong DNA (gray lines) with different $MgCl_2$ concentrations. B) Fluorescence counts of Armstrong (color lines) and Clone13 DNA (gray lines) with different $MgCl_2$ concentrations.
