# Supplementary figures and images for: Sequence-specific detection of different strains of LCMV in a single sample using tentacle probes
Source: Virol J. 2017 Oct 13;14:197. doi: 10.1186/s12985-017-0863-9 (PMC5640925; doi:10.1186/s12985-017-0863-9)

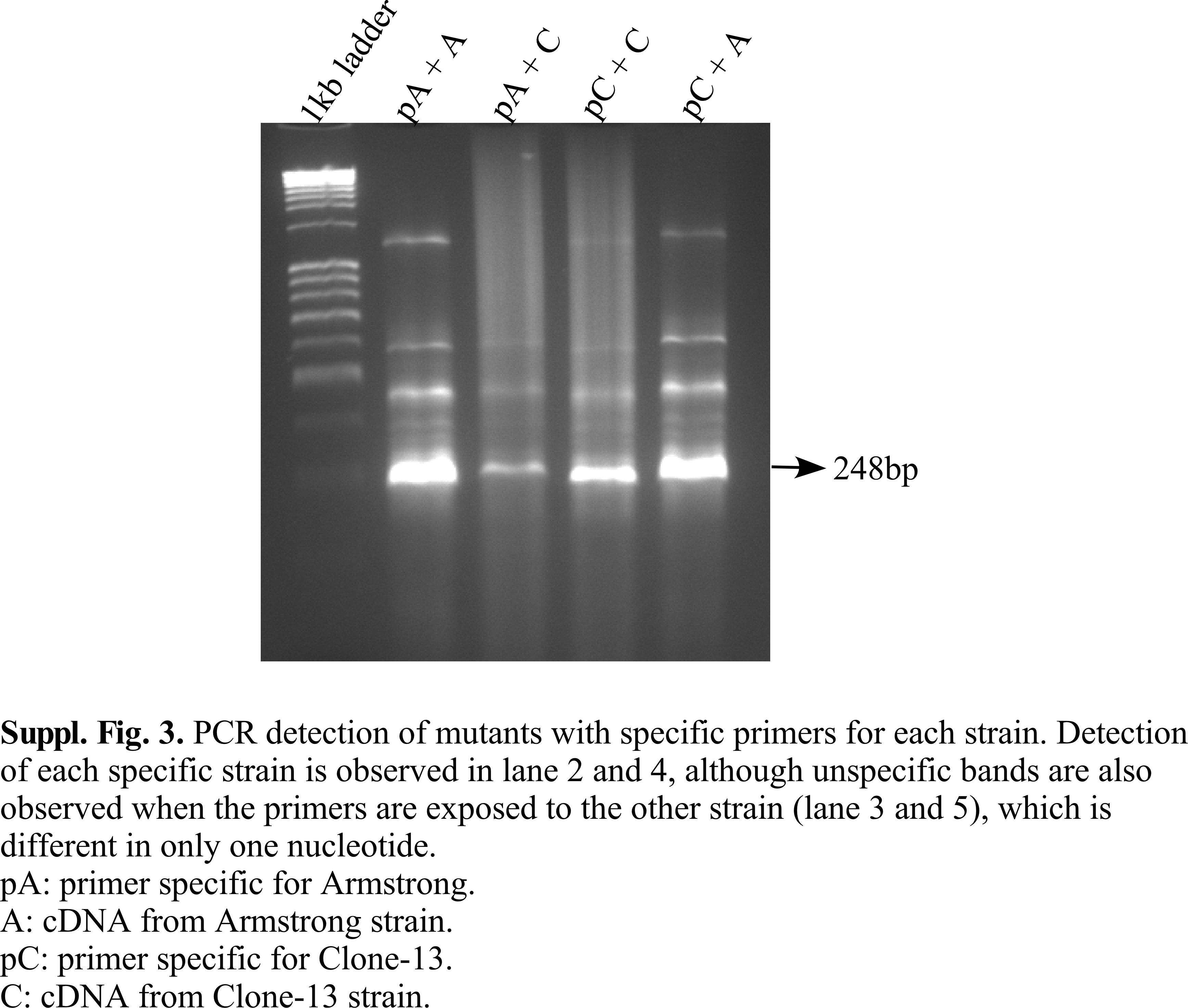

Supplement: Supplementary file 3 — PCR detection of mutants with specific primers for each strain. Detection of each specific strain is observed in lane 2 and 4, although unspecific bands are also observed when the primers are exposed to the other strain (lane 3 and 5), which is different in only one nucleotide. (TIFF 1377 kb) [file 12985_2017_863_MOESM3_ESM.tif]
